# P05.36. How do lay people conceptualise and reason about the use of placebos in healthcare?

**DOI:** 10.1186/1472-6882-12-S1-P396

**Published:** 2012-06-12

**Authors:** F Bishop, A Adams, E Aizlewood, G Lewith

**Affiliations:** 1 University of Southampton, Southampton, United Kingdom; 2 Northern Arizona University, Flagstaff, USA

## Purpose

Despite the prevalence and ethically contentious nature of the use of placebos in clinical practice and clinical trials, few studies have explored the perspectives of the general public. Our aim was to identify how lay people conceptualise and reason around the use of placebos in healthcare.

## Methods

Eleven focus groups were held with adult volunteers. Participants were purposefully recruited from rural and urban areas in the Midlands and South of England. They came from a range of walks of life, including students, professionals, home-makers, and retired people. Inductive thematic analysis was facilitated by Atlas.ti.

## Results

All participants recognised the term ‘placebo’ and exhibited diverse opinions as to how the placebo effect works and whether the placebo effect even exists. Honest doctor-patient communication was highly valued by participants, who typically saw placebo prescriptions as deceitful and therefore wrong. However, they also expressed the belief that that deception is necessary for the patient to experience potentially beneficial placebo effects. A pragmatic orientation was exhibited, wherein participants argued that if a placebo “works” then it is acceptable for a doctor to prescribe it. Placebos were considered more acceptable in certain circumstances, including self-limiting illness (e.g. common cold) and clinical trials (compared to clinical practice).

## Conclusion

Both a pragmatic orientation and an understanding of mind-body healing mechanisms seem to facilitate greater acceptance of placebos in medical research and clinical practice. Seeing deception as necessary to elicit placebo effects seems to prompt ethical concerns about the use of placebo in clinical practice. The views of the general public should be taken into account when researchers and doctors consider using placebos; these findings could be used to inform the development of more acceptable practices related to the use of placebos.

